# Effect of the Essential Oils of *Bursera morelensis* and *Lippia graveolens* and Five Pure Compounds on the Mycelium, Spore Production, and Germination of Species of *Fusarium*

**DOI:** 10.3390/jof8060617

**Published:** 2022-06-09

**Authors:** Yoli Mariana Medina-Romero, Mario Rodriguez-Canales, Marco Aurelio Rodriguez-Monroy, Ana Bertha Hernandez-Hernandez, Norma Laura Delgado-Buenrostro, Yolanda I. Chirino, Tonatiuh Cruz-Sanchez, Carlos Gerardo Garcia-Tovar, Maria Margarita Canales-Martinez

**Affiliations:** 1 Laboratorio de Farmacognosia, Unidad de Biología, Tecnología y Prototipos (UBIPRO), Facultad de Estudios Superiores Iztacala, Universidad Nacional Autónoma de México, Av. de los Barrios No. 1, Los Reyes Iztacala, Tlalnepantla de Baz CP 54090, Estado de Mexico, Mexico; ymmr727@gmail.com (Y.M.M.-R.); ana.b.hdez@hotmail.com (A.B.H.-H.); 2 Laboratorio de Investigación Biomédica en Productos Naturales, Carrera de Medicina, Facultad de Estudios Superiores Iztacala, Universidad Nacional Autónoma de México, Avenida de los Barrios Numero 1, Colonia Los Reyes Iztacala, Tlalnepantla de Baz CP 54090, Estado de Mexico, Mexico; mario.rodcan09@gmail.com (M.R.-C.); dr.marcorodriguezmonroy@gmail.com (M.A.R.-M.); 3 Laboratorio 10, Carcinogénesis y Toxicología, Unidad de Biomedicina (UBIMED), Facultad de Estudios Superiores Iztacala, Universidad Nacional Autónoma de México, Avenida de los Barrios Numero 1, Colonia Los Reyes Iztacala, Tlalnepantla de Baz CP 54090, Estado de Mexico, Mexico; nlbuenrostro@gmail.com (N.L.D.-B.); chirino@unam.mx (Y.I.C.); 4 Laboratorio de Servicio de Análisis de Propóleos (LASAP), Facultad de Estudios Superiores Cuautitlán, Universidad Nacional Autónoma de México, Av. Teoloyucan Km 2.5, San Sebastian Xhala, Cuautitlán Izcalli CP 54714, Estado de Mexico, Mexico; tonatiuh86@hotmail.com; 5 Laboratorio de Morfología Veterniaria y Biología Celular, Facultad de Estudios Superiores Cuautitlán, Universidad Nacional Autónoma de México, Av. Teoloyucan Km 2.5, San Sebastian Xhala, Cuautitlán Izcalli CP 54714, Estado de Mexico, Mexico; cgarciatov@yahoo.com.mx

**Keywords:** Burseraceae, Verbenaceae, medicinal plants, *Fusarium*, monoterpenes, antifungal activity, hyphal morphology, spore inhibition

## Abstract

The genus *Fusarium* causes many diseases in economically important plants. Synthetic agents are used to control postharvest diseases caused by *Fusarium*, but the use of these synthetic agents generates several problems, making it necessary to develop new alternative pesticides. Essential oils can be used as a new control strategy. The essential oils of *Bursera morelensis* and *Lippia graveolens* have been shown to have potent antifungal activity against *Fusarium*. However, for the adequate management of diseases, as well as the optimization of the use of essential oils, it is necessary to know how essential oils act on the growth and reproduction of the fungus. In this study, the target of action of the essential oils of *B. morelensis* and *L. graveolens* and of the pure compounds present in the essential oils (carvacrol, *p*-cymene, α-phellandrene, α-pinene, and Υ-terpinene) was determined by evaluating the effect on hyphal morphology, as well as on spore production and germination of three *Fusarium* species. In this work, carvacrol was found to be the compound that produced the highest inhibition of radial growth. Essential oils and pure compounds caused significant damage to hyphal morphology and affected spore production and germination of *Fusarium* species.

## 1. Introduction

The genus *Fusarium* includes many fungi pathogenic to plants. Species of the genus *Fusarium* produce numerous secondary metabolites associated with plant disease. Plant diseases caused by *Fusarium* have a great economic impact. Many plants are known to have at least one *Fusarium*-associated disease. Of the economically important plants listed by the American Phytopathological Society, more than 80% have at least one *Fusarium* disease [1]. Currently, for postharvest diseases caused by a variety of phytopathogenic fungi, synthetic agents are used repeatedly, which brings about various problems, such as environmental contamination, exceeding the restricted maximum residue limits on food products that generate problems in human health, in addition to the appearance of pathogen populations resistant to fungicides, among other adverse effects [2,3,4,5,6,7,8]. For example, when thiabendazole, imazalil, and *o*-phenylphenol are used in postharvest treatments, these compounds were shown to cause pathogens to develop some resistance [3,4]. Due to the possible toxicological risks involved in using synthetic agents, as well as the increased social and economic impact caused by fungal diseases, many synthetic agents have been retired from the market, which makes it necessary to produce safe food and, therefore, consequently, develop alternative pesticides [2,3,4]. The development of new alternative pesticides can help to reduce the negative impact of synthetic agents [2]. Alternative pesticides can be used to control diseases caused by *Fusarium*, considering many factors such as crop species, season, and pest complexity [9]. Among the new alternative pesticides, essential oils can be an alternative to the synthetic agents currently used to control phytopathogenic fungi because they constitute a source of novel bioactive substances [2,4]. Essential oils act in different ways on various types of complex diseases, so they can be applied in the food and agricultural industry in the same way as other synthetic agents [2].

The essential oils of plants used as natural products cover a wide spectrum of anti-fungal properties, different mechanisms of action, and limited side effects on the environment, and human health, so their use is appropriate to control *Fusarium* diseases [10]. Essential oils contain volatile restricted distribution metabolites produced by plants, which protect them from biotic factors such as pathogens, as well as abiotic factors such as excessive solar radiation [11].

Many studies have confirmed the strong antifungal activity of essential oils with a broad spectrum of inhibition. These antifungal agents have high volatility, are highly bio-degradable, have low residue generation, have low toxicity, and are safe for nontarget organisms [2,10]. Most of the chemical constituents of plant essential oils are terpenoid compounds, including monoterpenes. These low-molecular-weight compounds give them antifungal activity [12]. The essential oils of the species *Bursera morelensis* and *Lippia graveolens* have in vitro and in vivo antifungal activity [13,14,15,16]. In a previous study, the chemical composition of both essential oils was analyzed, and the main components were identified. In the same work, it was shown that these essential oils are not persistent during the postharvest control of *Fusarium* species [15]. However, the antifungal activity of essential oils and pure compounds has not been compared, nor has the effect of essential oils and pure compounds on the mycelium and spores of some *Fusarium* species been studied.

The purpose of this study was to compare the effect of essential oils of *B. morelensis* and *L. graveolens* and five pure compounds present in both essential oils, carvacrol, *p*-cymene, α-phellandrene, α-pinene, and Υ-terpinene, as well as to determine the target of action of these compounds by evaluating their effect on hyphal morphology and on the production and germination of spores of three *Fusarium* species.

## 2. Materials and Methods

### 2.1. Essential Oils and Pure Compounds

The essential oils (EOs) of the plants *B. morelensis* (voucher IZTA 42123) and *L. grave-olens* (voucher MCM8) were obtained by the hydrodistillation method. Subsequently, the essential oils were analyzed by gas chromatography/mass spectrometry (GC/MS) [15]. Of the essential oil components, five pure compounds present in one or both essential oils were selected. The selected pure compounds were carvacrol (CAS: 499-75-2, PubChem CID: 10364); *p*-cymene (CAS: 99-87-6, PubChem CID: 7463); α-phellandrene (CAS: 99-83-2, PubChem CID: 7460); α-pinene (CAS: 80-56-8, PubChem CID: 6654); and Υ-terpinene (CAS: 99-85-4, PubChem CID: 7461). All pure compounds were purchased from Sigma-Aldrich (St. Louis, MO, USA).

### 2.2. In Vitro Antifungal Activity of Pure Compounds

The strains *Fusarium sporotrichioides* ATTC NRLL3299, *Fusarium moniliforme* CDBB-H-265, and *Fusarium solani* CDBB-1407 were used. The strain of *F. sporotrichioides* was donated by the Laboratory of Plant Physiology at the FES Iztacala, UNAM, Tlalnepantla, Estado de Mexico, Mexico. The strains of *F. moniliforme* and *F. solani* were purchased from the CINVESTAV strain collection (Mexico D.F., Mexico). These strains were selected because they belong to the genus *Fusarium*, which has a tremendous economic impact on agriculture and produces many plant diseases [1]. Additionally, in the previous study of our research group, it was observed that they rapidly developed symptoms on a plant model and were highly sensitive to essential oils, which made them suitable test microorganisms for the study [15].

In a previous study conducted by our research group, the in vitro antifungal activity of the essential oils of *B. morelensis* and *L. graveolens* was evaluated [15], so in the present work, the antifungal activity in vitro of the five pure compounds present in both essential oils was measured to later compare the effect produced by the essential oils and the pure compounds. The radial growth inhibition method was used to determine the medium fungicidal concentration (CF_50_). The concentrations of carvacrol, *p*-cymene, α-phellandrene, α-pinene, and Υ-terpinene used were 0.25, 0.5, 1.0, 2.0, and 4.0 µL/mL, respectively. The compounds at varying concentrations were added with a micropipette to potato dextrose agar, mixed, and placed in a divided Petri dish. Three 0.5-mm diameter inocula of the same strain were placed equidistantly in each compartment and incubated at 23 °C for 72 h. Subsequently, the colony area was measured, and colony growth inhibition was determined [15,17]. The experiment was performed in triplicate.

### 2.3. Activity of Essential Oils and Pure Compounds on Mycelium

In a previous study performed by our research group, essential oils were shown to have a potent antifungal effect on the growth of the three *Fusarium* species [15]; however, the way in which essential oils affect the growth of the mycelium is unknown. To observe the possible effect of essential oils and pure compounds on the mycelium structure of the three *Fusarium* species, fungi were exposed to essential oils and pure compounds. Two experiments were performed to determine the activity of essential oils and pure compounds on the mycelium of the three *Fusarium* species. In both experiments, the fungi were cultivated in 10 mL vials containing 5 mL of Sabouraud broth under static conditions at room temperature and with a fluorescent light/darkness photoperiod of 12:12 h. In each vial, five inocula of 3 mm in diameter were placed from the edge of cultures of each of the strains grown in potato dextrose agar for eight days of incubation. In the first experiment, two concentrations of 2.0 and 4.0 µL/mL of the essential oils and pure compounds were added to the fungi inoculated in the vials to grow exposed for 48 h. In the second experiment, the two concentrations of 2.0 and 4.0 µL/mL of each essential oil and pure compound were added to the fungi inoculated in the vials after 48 h of incubation. This experiment was monitored at five h. The vials were covered with Parafilm^®^ paper to prevent the compounds from volatilizing. For both experiments, when the incubation period ended, the mycelium was stained with calcofluor-white and propidium iodide; as the negative control, a culture growing without essential oils or pure compounds was used. Preparations were viewed on an SP8 LIGHTNING confocal microscope from Leica Microsystems (Wetzlar, Germany). Preparations were viewed at a total magnification of 100. The calcofluor-white dye was viewed at a wavelength of 450 nm, and the propidium iodide was viewed at a wavelength of 580 nm. All experiments were performed in triplicate [16,18].

### 2.4. Activity of Essential Oils and Pure Compounds on Spore Production and Germination

Using a plant model made by our research group in a previous experiment, essential oils were observed to inhibit the growth of the three *Fusarium* species growing on cherry tomatoes [15]; this inhibition could be because the essential oils influence spore production and germination. To determine the effect of essential oils and pure compounds on the spore production and germination of the three *Fusarium* species, spores were exposed to essential oils and pure compounds. To evaluate the activity of spore production and germination, essential oils and pure compounds were used at concentrations of 2.0 and 4.0 μL/mL [19]. Previously, suspensions of the spores of each strain of fungus with 2.2 × 10^5^ spores mL^−1^ were made. Based on this information, aliquots of 100 μL of the spore suspension of each of the strains were placed in vials with sterile distilled water. One of the concentrations of each essential oil or pure compound was added to the vials with the spore suspensions, and they were allowed to incubate under static conditions at room temperature and with a fluorescent light/darkness photoperiod of 12:12 h for 24 h or 48 h, depending on fungal growth. Subsequently, the vial was observed under a microscope to determine the production and germination of spores. The total number of spores was counted, together with the spores that generated germ tubes, and the percentages of inhibition and germination were calculated. As a negative control, suspensions of distilled water without essential oils or pure compounds were employed. The experiments were performed in triplicate [2].

### 2.5. Statistical Analysis

The mean and standard deviation of the experiments were determined. Analysis of variance (ANOVA) was performed to test for significant differences (*p* < 0.05) with Tukey’s honestly significant difference (HSD) multiple comparison test using the GraphPad Prism version 7 program. The CF_50_ values were determined by probit analysis based on the percentage of inhibition obtained for each concentration tested using the same program [15,18,20].

## 3. Results

### 3.1. In Vitro Antifungal Activity of Pure Compounds

**Figure 1:**
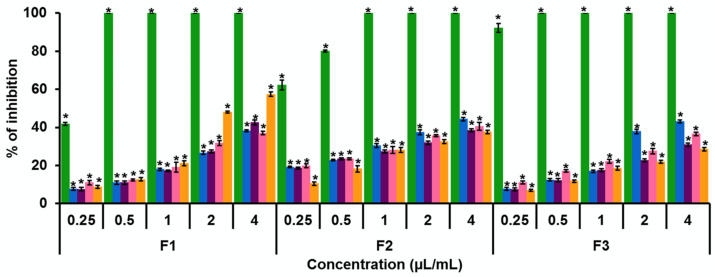
Effect of five pure compounds on the growth of three *Fusarium* strains. Percentage of growth inhibition in an in vitro model of *Fusarium sporotrichioides* (F1), *Fusarium moniliforme* (F2), and *Fusarium solani* (F3) exposed to carvacrol (green), *p*-cymene (blue), α-phellandrene (purple), α-pinene (pink), and Υ-terpinene (orange) at concentrations of 0.25, 0.50, 1.0, 2.0, and 4.0 µL/mL. * Statistically significant values of mean with respect to the control (*p* < 0.0001).

Figure 1 shows the percentages of inhibition of radial growth of fungi by the five pure compounds at concentrations of 0.25 to 4.0 μL/mL. Except for carvacrol, the essential oils showed greater inhibition of the radial growth of the three *Fusarium* species. *F. solani* was the most affected by essential oils as well as by carvacrol, from a concentration of 0.25 μL/mL. However, *F. sporotrichioides* and *F. moniliforme* were completely inhibited by *L. graveolens* essential oil at a concentration of 4.0 μL/mL. At a concentration of 0.25 μL/mL, the two essential oils inhibited fungal growth at values higher than 45% [15]. Regarding the compounds, all pure compounds exhibited concentration-dependent inhibition. Carvacrol was the compound that caused the highest growth inhibition of the three fungi. From a concentration of 0.50 μL/mL, the same or higher than 80% inhibition was observed in all fungi. From a concentration of 1.0 μL/mL, the growth of the three strains was completely inhibited. The most affected fungus was *F. solani*. The CF_50_ values for *F. sporotrichioides*, *F. moniliforme*, and *F. solani* were 0.25, 0.19, and 0.02 μL/mL, respectively. The *p*-cymene and the α-pinene inhibited the growth of the fungi in values higher than 35% at the maximum test concentration. *F. moniliforme* was the fungus most affected by both compounds. The CF_50_ values for *p*-cymene for *F. sporotrichioides*, *F. moniliforme*, and *F. solani* were 5.65, 4.28, and 4.89 μL/mL, respectively. The CF_50_ values for α-pinene against *F. sporotrichioides*, *F. moniliforme*, and *F. solani* were 5.91, 15.82, and 16.09 μL/mL, respectively. α-Phellandrene produced an inhibition higher than 30% at a concentration of 4.0 μL/mL. This compound affected more *F. sporotrichioides*. The CF_50_ values for α-phellandrene for *F. sporotrichioides*, *F. moniliforme*, and *F. solani* were 8.44, 5.20, and 10.68 μL/mL, respectively. Finally, Υ-terpinene inhibited growth at values higher than 25%. However, in *F. sporotrichioides*, the inhibition was higher than 45% from the concentration of 2.0 μL/mL, so this was the fungus most affected by this compound (Figure 1). The CF_50_ values for Υ-terpinene in *F. sporotrichioides*, *F. moniliforme*, and *F. solani* were 2.71, 9.02, and 21.04 μL/mL, respectively.

In the evaluation of both the essential oils of *B. morelensis* and *L. graveolens* and the pure compounds, concentration-dependent inhibition was observed. However, in a previous study with essential oils, at a concentration of 0.5 μL/mL, the inhibition was found to be higher than 60% for both oils. The CF_50_ for the essential oil of *B. morelensis* for the fungi *F. sporotrichioides*, *F. moniliforme*, and *F. solani* was 0.27, 0.31, and 0.24 μL/mL, respectively, and for the essential oil of *L. graveolens* was 0.15, 0.17, and 0.20 μL/mL, respectively [15]. The CF_50_ values for carvacrol were 0.25, 0.19, and 0.02 μL/mL, respectively. Carvacrol has values similar to the values of the essential oils. However, the CF_50_ of each compound was evaluated individually, except for carvacrol; the values were between 2.71 and 21.04 μL/mL.

### 3.2. Activity of Essential Oils and Pure Compounds on Mycelium

**Figure 2:**
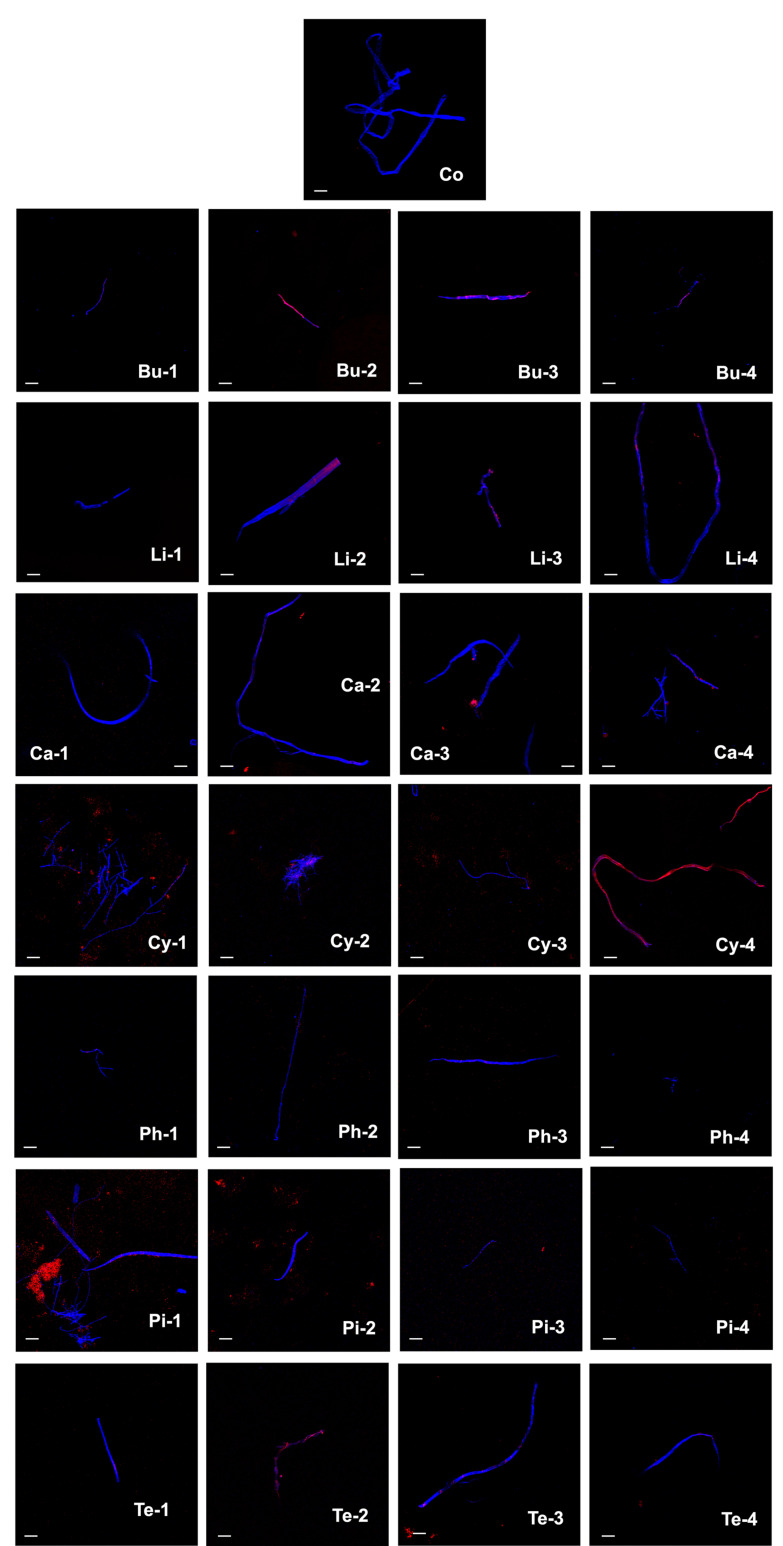
Effect of essential oils and five pure compounds on the mycelium of *Fusarium solani*. Microphotographs were obtained by fluorescence microscopy and dyed with calcofluor-white (ʎ = 450 nm) and propidium iodide (ʎ = 580 nm) at a total magnification of 100. The scale-bar represents 50 μm. Control (Co), *Bursera morelensis* essential oil (Bu), *Lippia graveolens* essential oil (Li), carvacrol (Ca), *p*-Cymene (Cy), α-phellandrene (Ph), α-pinene (Pi), and Υ-terpinene (Te). The numbers correspond to the concentration of 2.0 µL/mL at 4 h (1) and at 48 h (2) of exposure and at the concentration of 4.0 µL/mL at 4 h (3) and at 48 h (4) of exposure.

Figure 2 shows the effect of essential oils and pure compounds on the mycelium of *F. solani*. In the essential oil of *B. morelensis*, at a concentration of 2.0 μL/mL at 4 h of exposure, propidium iodide is not observed to penetrate the hyphae, so it is possible that such severe damage has not yet occurred, while at 48 h, as well as the concentration of 4.0 μL/mL at 4 h and 48 h of exposure, the essential oil already penetrated the hyphae of *F. solani*. In the essential oil of *L. graveolens*, at a concentration of 2.0 μL/mL at 4 h of exposure, areas without color can be seen in the hyphae, which indicates damage to this structure, at the same concentration but at 48 h, and at 4 μL/mL at 4 h and 48 h of exposure, penetration of the propidium iodide dye is observed, which also indicates damage to the hyphae.

In carvacrol, at a concentration of 2.0 μL/mL at 4 h and 48 h of exposure, areas of fainter calcofluor-white staining were observed; however, the penetration of propidium iodide in the hyphae was not observed. However, at a concentration of 4.0 μL/mL at 4 h and 48 h of exposure, calcofluor-white dye staining was observed in the hyphae, and in the periphery, some circles stained with propidium iodide were observed, which may indicate that there was leakage of hyphal content. In *p*-cymene, at concentrations of 2.0 μL/mL at 4 h and 48 h of exposure, broken and aggregated hyphae are observed in these treatments, as well as in 4.0 μL/mL at 4 h of exposure. The same effect is observed as in carvacrol, hyphae stained with calcofluor-white, and in the periphery circles stained with propidium iodide, so there is also leakage of the content of the hyphae. However, at a concentration of 4.0 μL/mL at 48 h of exposure, the highest damage produced by both essential oils and pure compounds was observed, since the hyphae were almost completely stained by propidium iodide dye and only a small amount of calcofluor-white staining was observed. The α-phellandrene, at concentrations of 2.0 and 4.0 μL/mL at 4 h of exposure, as well as the concentration of 2.0 μL/mL at 48 h of exposure, showed greater penetration of the calcofluor-white dye, and a small amount of propidium iodide staining was also observed; however, at the concentration of 4.0 μL/mL at 48 h of exposure, only calcofluor-white staining was observed, as well as a deformation in the morphology of the hyphae. In α-pinene, the staining of the hyphae with calcofluor-white and outside regions stained with propidium iodide can be seen at concentrations of 2.0 μL/mL at 4 h and 48 h of exposure. Additionally, at a concentration of 2.0 μL/mL at 4 h of exposure, broken and aggregated hyphae were observed, as in *p*-cymene. At a concentration of 4.0 μL/mL at 4 h of exposure, red circles are observed both outside and inside the hypha, and at the same concentration but at 48 h of exposure, areas unstained by calcofluor-white are seen inside the hypha. Finally, with Υ-terpinene, at a concentration of 2.0 μL/mL at 4 h of exposure, only calcofluor-white staining was observed at the same concentration, but at 48 h of exposure, greater staining by propidium iodide was observed, as well as deformations in the morphology of the hyphae. However, at the concentration of 4.0 μL/mL at 4 h and 48 h of exposure, staining was observed for both calcofluor-white and propidium iodide and unstained regions by calcofluor-white within the hypha (Figure 2).

Both the essential oils and the pure compounds damage the cell wall and the cell membrane since when comparing the microphotographs, in most of the treatments, the staining is observed to be appreciated not only of calcofluor-white but also of propidium iodide, unlike the microphotograph of the control, which only shows calcofluor-white staining. In general, essential oils cause more damage than pure compounds. However, it is important to highlight the effect of *p*-cymene at a concentration of 4.0 μL/mL at 48 h and of Υ-terpinene at a concentration of 2.0 μL/mL at 48 h. In both cases, higher damage was observed because of the coloration produced by propidium iodide dominates over the coloration of calcofluor-white (Figure 2).

### 3.3. Activity of Essential Oils and Pure Compounds on Spore Production and Germination

**Figure 3:**
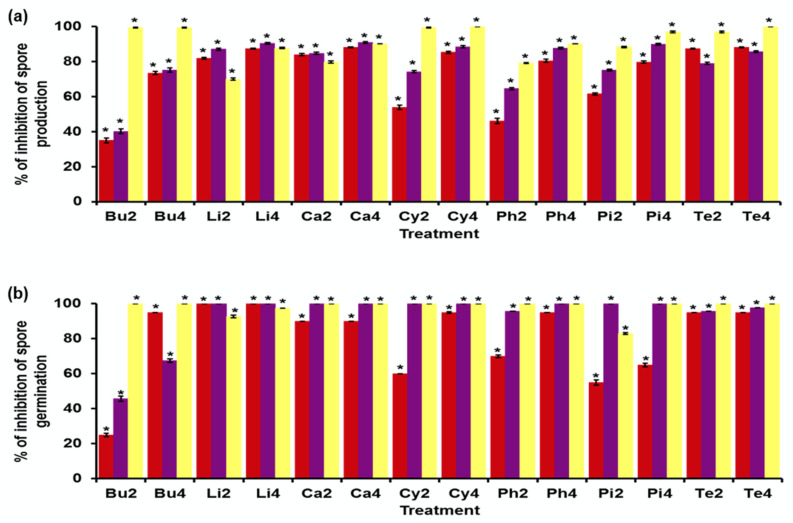
Effect of essential oils and five pure compounds on spore production and germination of three *Fusarium* strains. (**a**) Percentage of inhibition of spore production. (**b**) Percentage of inhibition of spore germination. *Fusarium sporotrichioides* (red), *Fusarium moniliforme* (purple), and *Fusarium solani* (yellow) exposed to *Bursera morelensis* essential oil (Bu), *Lippia graveolens* essential oil (Li), carvacrol (Ca), *p*-cymene (Cy), α-phellandrene (Ph), α-pinene (Pi), and Υ-terpinene (Te). The numbers correspond to the concentrations of 2.0 µL/mL (2) and 4.0 µL/mL (4). * Statistically significant values of mean with respect to the control (*p* < 0.0001).

Figure 3 shows the effect of essential oils and pure compounds on spore production (Figure 3a) and germination (Figure 3b) of the three *Fusarium* strains. In general, concentration-dependent inhibition of spore production and germination is observed.

In the inhibition of spore production (Figure 3a), *F. sporotrichioides* presented inhibition values higher than 70% at a concentration of 4.0 μL/mL. The highest inhibition is produced by carvacrol and Υ-terpinene. In the case of *F. moniliforme*, the inhibition was similar to or higher than 75% at a concentration of 4.0 μL/mL. The highest inhibition was produced by *L. graveolens* essential oil and carvacrol. Finally, *F. solani* presents an inhibition similar to or higher than 69% at the two concentrations. *p*-Cymene and Υ-terpinene are the only compounds that completely inhibit spore production. *F. solani* is the most affected by both essential oils and pure compounds.

Regarding spore germination (Figure 3b), *F. sporotrichioides* presented inhibition values like or higher than 65% at a concentration of 4.0 μL/mL in all essential oils and pure compounds. *L. graveolens* essential oil completely inhibited spore germination at both concentrations evaluated. The germination of *F. moniliforme* spores was inhibited in values higher than 65% at a concentration of 4.0 μL/mL. *L. graveolens* essential oil, carvacrol, *p*-cymene, and α-pinene completely inhibited spore germination at both concentrations. In addition, α-phellandrene completely inhibited spore germination at a concentration of 4.0 μL/mL. The inhibition of the germination of *F. solani* spores was found in values higher than 80% in the two concentrations evaluated. *B. morelensis* essential oil, carvacrol, *p*-cymene, α-phellandrene, and Υ-terpinene completely inhibited spore germination at both test concentrations. However, α-pinene completely inhibited spore germination only at a concentration of 4.0 μL/mL. Again, *F. solani* is the fungus most affected by both essential oils and pure compounds.

## 4. Discussion

Generally, the essential oils have higher antifungal activity than pure compounds independently evaluated. The differences in the antifungal activity of essential oils and pure compounds are related to the active compounds [10]. Some of the compounds present in essential oils are phenolic compounds, terpenes, alcohols, and aldehydes [7,15]. Therefore, the antifungal activity of essential oils depends on the combination and proportions of different compounds found in their composition, as well as their synergistic interactions [6,21,22]. The synergistic effect of some minor compounds, together with the major components, can also contribute to the fungicidal activity of essential oils. Compounds such as phenols and aldehydes may be responsible for severe membrane damage, resulting in high fungicidal activity [10].

Among the chemical compounds present in the essential oils of *B. morelensis* and *L. graveolens*, terpenes are the major constituents, mainly monoterpenes [12,15,21]. Monoterpenes are low-molecular-weight compounds, so they easily diffuse through the cell membrane to induce biological reactions [12]. Previous studies confirm that the inhibitory effects on many fungi are attributed to low-molecular-weight components and highly lipophilic characteristics that result in their ability to easily penetrate the phospholipid membrane cells and lead to their structural disorder and permeability, allowing cytoplasmic leakage [7,12]. In addition to being major constituents of essential oils, many of them have reported biological activity [15,18,22]. For example, carvacrol was shown to have antifungal activity in a study with *Fusarium* sp. It completely inhibited the fungus mycelial growth [23]. In two other studies in which α-pinene was evaluated, it was found to have moderate antifungal activity because it shows low mycelial growth inhibition of *F. solani* [24] and *F. subglutinans* f.sp. *ananas* [25]. On the other hand, Υ-terpinene showed potent antifungal activity against the pathogens *F. subglutinans*, *F. cerealis*, *F. verticillioides*, *F. proliferatum*, *F. oxysporum*, *F. sporotrichioides*, *Aspergillus tubingensis*, *A. carbonarius*, *Alternaria alternata*, and *Penicillium* sp [26], and against *F. culmorum*, *F. graminearum*, and *Pyrenophora graminea* [27]. In another study, in which α-pinene, *p*-cymene, and Υ-terpinene were used against *F. oxysporum*, *A. mali*, *Botrytis cinerea*, and *Verticillium dahliae*, the compounds inhibited growth of all pathogenic fungi. Furthermore, *p*-cymene had higher activity than the other two compounds, and the most affected fungus was *F. oxysporum* [28]. Finally, another study evaluated carvacrol, thymol, α-pinene, and Υ-terpinene for postharvest control of *A. alternata* in *Ziziphus jujuba* Mill. In this work, carvacrol and thymol were the most effective antifungal compounds. However, α-pinene and Υ-terpinene showed moderate to weak antifungal activity against the pathogen *A. alternata* [7]. In the two studies in which three or more pure compounds were used, they were tested against different phytopathogenic fungi that cause significant crop losses, including a species of *Fusarium.* In all cases, the pure compounds were shown to act against a broad spectrum of phytopathogenic fungi by inhibiting mycelial growth.

With the micrographs of the present work, the lipophilic nature of the pure compounds was demonstrated since they can penetrate the cell membrane, causing severe damage and even death to the fungal cells. The lipophilic nature of the pure compounds plays a crucial role in antifungal activity. The target of action of pure compounds, in general, was also determined since they cause structural and functional damage by disrupting membrane permeability and the osmotic balance of the cell. This disruption of ion transport processes indicates that carvacrol interacts with membrane proteins and other cellular components [8].

Calcofluor white has an affinity for chitin and cellulose; therefore, it stains fungal cell wall chitin [29,30]. Damage to fungal cells is seen when calcofluor-white dye differentially stains some areas of the hyphae. This dye also allows us to clearly see another effect of the pure compounds on the hyphae of *F. solani* since broken and aggregated hyphae are also seen in the micrographs, which is one of the effects reported by monoterpenes in previous work [22].

The cell membrane is a selectively permeable barrier between the cellular content and the environment. When propidium iodide enters the cell, it binds to the nucleic acids and increases their fluorescence, so when the cells have damaged membranes, they fluoresce red, and therefore they are considered dead cells. However, when the membranes are intact, they do not fluoresce red and are considered living cells. However, when the dye stains the hyphae, the cells have lost their permeability barrier, which represents irreparable damage and, therefore, cell death [31,32]. The hyphae that did not stain with propidium iodide do not have apparent damage to the cell membrane because, in viable cells, the membrane remains intact and does not allow the passage of propidium iodide [31]. In a study carried out with carvacrol against *Colletotrichum fructicola*, carvacrol was found to increase the permeability of the cell membrane of *C. fruticola*, allowing the leakage of carbohydrates, proteins, and nucleic acids from the cell. In addition, carvacrol led to the development of abnormalities in the morphology, disruption of cytoplasmic membranes, and degradation of intracellular organelles. The effect observed in this work can be attributed to the ability of carvacrol to damage the cell membrane, resulting in loss of osmotic balance and leakage of cellular components, eventually leading to cell death [32].

In a previous study by our research group, the effect of the essential oils of *B. morelensis* and *L. graveolens* on the germination of spores of *F. sporotrichioides*, *F. moniliforme*, and *F. solani* was demonstrated by means of an experiment in a plant model and on the inhibition of the growth of the mycelium of the three species. In all cases, concentration-dependent inhibition was found. Additionally, the chemical composition of the essential oils was determined [15]. However, in this previous study, pure compounds present in essential oils had not been included, nor had experiments been carried out quantitatively and at the microscopic level on spores, nor had the effect of essential oils or pure compounds been observed on the hyphae of the three *Fusarium* species. In the study carried out with α-pinene against *F. circinatum*, it was observed that this compound reduced spore germination and mycelial growth. However, α-pinene has a higher effect on mycelial growth than spore germination [33]. In another study, the effect of the essential oil of *Bunium persicum* and pure compounds against strains of *F. verticillioides* was evaluated. In this work, it was found that both the essential oil and the pure compounds have potent antifungal activity since they inhibit mycelial growth and inhibit spore germination. The inhibition of spore germination and mycelial growth was concentration-dependent [34]. In work carried out with the essential oil of *Rosmarinus officinalis* against *F. verticillioides*, it was observed that the essential oil reduced mycelial growth and spore production [35]. On the other hand, the work evaluated four essential oils against *F. oxysporum* f.sp. *lycopersici*, all essential oils, inhibited mycelial growth and spore germination [36]. Finally, in the study of 38 essential oils against *F. avenaceum*, *Aphanomyces euteiches*, *Botrytis cinerea*, *Colletotrichum lentis*, *Didymella pisi*, *D. rabiei*, *D. lentis*, *Stemphylium beticola*, *Sclerotinia sclerotiorum*, and *Pythium sylvaticum*, it was found that all essential oils inhibited the mycelial growth. Seven of these essential oils were selected to see their effect on the spore germination of *F. avenaceum*, *B. cinerea*, and *D. pisi*, and it was observed that the thyme essential oil completely inhibited the spore germination of *F. avenaceum* and *D. pisi* [37].

In other works, in which essential oils are used to evaluate their effect on the mycelial growth and spore germination of different species of phytopathogens, similar results to those previously mentioned are observed when *Fusarium* species were used; that is, essential oils affect both mycelial growth and spore germination, generally in a concentration-dependent manner. For example, a concentration-dependent reduction in spore was used in a study where seven essential oils from various plants were used against *Aspergillus niger*, *Aspergillus oryzae*, *Aspergillus ochraceus*, and germination was observed, as well as inhibition of mycelial growth [10]. In work carried out with 14 essential oils extracted from Uruguayan native plants used in the postharvest control of *Phyllosticta citricarpa*, all the essential oils were found to have at least one monoterpene; among the most abundant compounds, α-pinene was identified, and all essential oils were shown to reduce spore production and inhibit mycelial growth of *P. citricarpa* [5]. In another work where four pure compounds were used to evaluate the antifungal activity against *Aspergillus flavus*, *A. niger*, and *A. ochraceus*, in the presence of the evaluated compounds, the production of spores was shown to be reduced, in addition to the growth of the fungal mycelium being inhibited [22].

Based on the experiments of the studies previously mentioned, in general, essential oils and pure compounds are found to inhibit spore production and germination along with mycelial growth. Therefore, the target of action of the inhibition of spore production and germination, in the same way as the inhibition of mycelial growth, is associated with the destruction of the fungal cell membrane system, which leads to the disruption of cytoplasmic membranes and degradation of intracellular organelles and is caused by the penetration of pure compounds into the cell membrane [10,32].

## 5. Conclusions

Carvacrol was the compound that had the highest radial growth inhibition of the three *Fusarium* species. The essential oils of *B. morelensis* and *L. graveolens* and the pure compounds (carvacrol, *p*-cymene, α-phellandrene, α-pinene, and Υ-terpinene) cause severe damage to *F. solani* hyphae. The highest inhibition of spore production is produced by *L. graveolens* essential oil, carvacrol, *p*-cymene, and Υ-terpinene. The essential oils and pure compounds produce potent inhibition of spore germination. Inhibition of radial growth and spore production and germination was concentration-dependent.

## Data Availability

Not applicable.
